# An Exploratory Study of Intensive Neurofeedback Training for Schizophrenia

**DOI:** 10.1155/2017/6914216

**Published:** 2017-06-21

**Authors:** Wenya Nan, Feng Wan, Lanshin Chang, Sio Hang Pun, Mang I. Vai, Agostinho Rosa

**Affiliations:** ^1^Department of Psychology, College of Education, Shanghai Normal University, Shanghai, China; ^2^Department of Electrical and Computer Engineering, Faculty of Science and Technology, University of Macau, Macau; ^3^Department of Psychology, Faculty of Social Sciences and Humanities, University of Macau, Macau; ^4^State Key Laboratory of Analog and Mixed Signal VLSI, University of Macau, Macau; ^5^LaSEEB-System and Robotics Institute, Department of Bioengineering, Instituto Superior Tecnico, University of Lisbon, Lisbon, Portugal

## Abstract

Schizophrenia is a chronic and devastating brain disorder with ongoing cognitive, behavioral, and emotional deteriorated functions. Neurofeedback training, which enables the individuals to regulate their brain activity using a real-time feedback loop, is increasingly investigated as a potential alternative intervention for schizophrenia. This study aimed to explore the effect of short but intensive neurofeedback training for schizophrenic patients with difficulty for long-time training. A middle-aged woman with chronic schizophrenia completed the intensive training of alpha/beta2 (20–30 Hz) in four consecutive days with a total training duration of 13.5 hours. The results showed that her alpha/beta2 increased over sessions, and her behavior performance including short-term memory, mood, and speech pattern was improved at the end of neurofeedback training. Importantly, a 22-month follow-up found a dramatic improvement in both positive and negative symptoms. These positive outcomes suggest that such intensive neurofeedback training may provide new insight into the treatment of schizophrenia and thus deserves further study to fully examine its scope.

## 1. Introduction

Schizophrenia is regarded as the most devastating brain disorder with ongoing cognitive, behavioral, and emotional deteriorated functions. The life qualities of the schizophrenic patients dramatically decrease. The traditional treatment for schizophrenia is antipsychotic medication. However, antipsychotic medications have side effects when used long term, which leave patients with limited and deteriorated functions. Moreover, antipsychotic medications do not treat negative symptoms effectively [1]. Therefore, current pharmacological treatment is insufficient and the development of new treatment options is necessary.

In recent decades, neurofeedback (NF) as an innovative noninvasive technique aiming at self-regulating the brain activity using a real-time feedback loop mechanism under operant control has been demonstrated more and more benefits on human brain functions and behavior performance for not only healthy people but also patients with a variety of diseases [2–5]. With respect to NF for schizophrenia, some researchers have investigated electroencephalography (EEG) NF as a potential nonpharmaceutical alternative for treatment of schizophrenia. During EEG NF, the EEG signal is recorded from the electrodes placed on human scalp and the relevant EEG components are extracted and fed back to the training individual using an online feedback loop in the form of audio, visual, or their combinations [6]. An earlier study in Gruzelier et al. [7] reported that 16 schizophrenic patients were able to learn control the left-right asymmetry in slow potential negativity at the sensory motor area (C3 and C4). Bolea [8] employed NF on more than 70 hospital inpatients with chronic schizophrenia and found improvements in the EEG patterns and in cognitive, affective, and behavioral patterns. Another study from Surmeli et al. [1] also reported positive effects on 51 inpatients by quantitative EEG- (qEEG-) guided NF training. Besides the EEG NF, with the development of modern real-time brain imaging technology, some studies investigated the feasibility of real-time functional magnetic resonance imaging (rtfMRI) NF in patients with schizophrenia [9–11]. In Ruiz et al. [10], nine schizophrenic patients learned self-control of the blood oxygenation level-dependent (BOLD) signal of the bilateral anterior insula cortex by rtfMRI NF, which led to changes in the perception of emotions and modulations of the brain network connectivity. In addition to anterior insula cortex, the BOLD signal of the anterior cingulate cortex (ACC) could also be successfully regulated by rtfMRI NF, which were associated with symptom enhancement in patients with schizophrenia [9, 11].

The aforementioned EEG NF studies which showed very promising results adopted larger session number, longer training process, and personalized training parameters [1, 8]. Bolea [8] suggested that enhancement of alpha and reduction of fast beta seemed very important in inpatients with chronic schizophrenia, but its process spread longer time and thus it did require patience, while for some schizophrenic patients, it is very difficult to perform the long term NF training. For example, patients who live home will be very difficult to follow NF training for a longer period of time. Thus, we wonder whether short but intensive NF training could also show benefits on patient with schizophrenia. A middle-aged woman with chronic schizophrenia was recommended and voluntarily participated in this study. The NF training was to increase the relative amplitude of alpha/beta2 (20–30 Hz) ratio within four consecutive days for a total training duration of 13.5 hours. Both immediate effect and long-lasting effect of a 22-month follow-up were examined.

## 2. Methods

### 2.1. Patient

The patient with chronic schizophrenia was a 51-year-old single woman. She received a prominent education and had a prestigious professional career. She did not have any head trauma or other medical problems, and she did not have drug or alcohol abuse. Both sides of her family members did not have any history of schizophrenia. Before NF training, she had suffered schizophrenia for more than seven years and was in hospital to treat her schizophrenic episodes for several times. After being released back home, she was treated as an incapable person who needed a 24-hour close guardianship for her daily routine from her family. Her illness made her give up her career and created a huge burden to herself and her family. Based on her own accounts, she did not take the antischizophrenia medications regularly due to various reasons, including the side effects from those antipsychotics (uncomfortable sensations, dizziness, and blurry vision), self-rejection of her psychosis, and suspicion of drug toxicity. She did not have noticeable improvement with either single antipsychotic treatment or combined antipsychotic and psychotherapeutic treatments. Thus, she was recommended by her psychotherapist to receive NF training to explore whether NF training could help her. She took olanzapine and clonazepam as usual before, during, and after NF sessions. The protocol was in accordance with the Declaration of Helsinki and approved by the local Research Ethics Committee (University of Macau), and informed written consent was obtained from the patient before NF training.

### 2.2. NF Training

The patient had auditory hallucination symptoms. Previous studies have reported that schizophrenics have reduced alpha amplitude and increased beta activity [12–15], while the clinical improvements in schizophrenic patients after antipsychotic treatment or transcranial magnetic stimulation (TMS) have been found to correlate with the increases in alpha power [16, 17]. Furthermore, Bolea [8] pointed out the importance of reduction of fast beta and enhancement of alpha at P4, PO4, and CP4 by NF training. Therefore, the training objective was to increase alpha amplitude simultaneously to decrease beta2 (20–30 Hz) amplitude at P4 location. The ground was located at the forehead, and the reference was placed on the left mastoid. The EEG was acquired by an EEG amplifier (Sommeil 800 from Meditron Eletromedicina Ltda, SP, Brazil) with an analog band-pass filter from 0.1 to 70 Hz. The sampling frequency was 256 Hz. Somnium software platform (Cognitron, SP, Brazil) with NF plugin (LaSEEB-ISR, Portugal) was utilized to record the EEG signal and show the NF interface. In the Somnium system, the signals were filtered by a band-pass filter from 0.5 to 30 Hz, and a notch filter at 50 Hz. Circuit impedance was kept below 10 kΩ for all electrodes [18].

**Figure 1:**
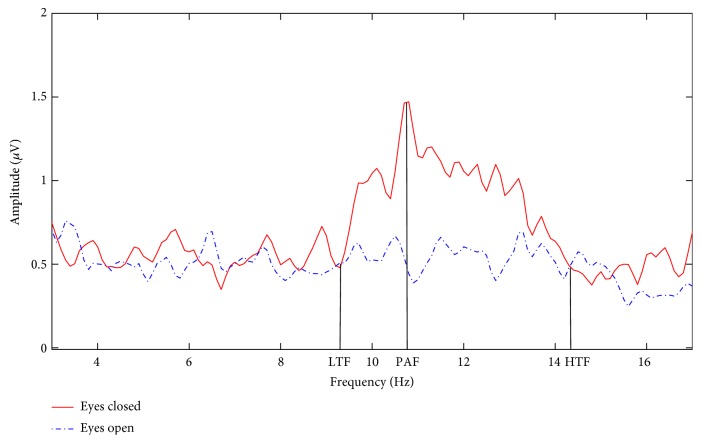
The illustration of determining the IAB.

Before NF, two 60-second EEG epochs during the resting state were recorded while the patient had her eyes open and closed, respectively. The feedback parameter was set to the relative amplitude of alpha/beta2 ratio at P4 location. Using the amplitude spectrum instead of the power spectrum prevents excessive skewing which results from squaring the amplitude and, thus, increases statistical validity [19]. The amplitude was calculated by FFT every 125 ms with a 2-second data window. Due to the large interindividual differences in alpha band, we utilized the individual alpha band (IAB) instead of the standard alpha band (8–12 Hz) [20].  Figure 1() illustrates an example on how to determine the IAB (LTF-HTF), in which low transition frequency (LTF) and high transition frequency (HTF) are the crossings of EEG amplitude spectrum in the eyes open and eyes closed resting state and peak alpha frequency (PAF) in the EEG amplitude spectrum in the eyes closed state separates the IAB into the lower IAB (LTF-PAF) and the upper IAB (PAF-HTF) [20].

The training process consisted of two parts. The first part was the user control sessions in which the patient adapted herself to the NF program and explored the mental activities. The session duration and session number were controlled by the patient. She could start and stop training and have a rest between session intervals. The mental strategy used was annotated after each user control session. As a result, the duration of each session varied from 1 to 15 minutes and the mean duration of each session was 7.5 minutes. The total training time of the first part was 12.5 hours in four consecutive days. More specifically, the total training duration was 1 hour and 20 minutes on day 1, 3 hours on day 2, 4 hours and 40 minutes on day 3, and 3.5 hours on day 4. By the user control sessions, 6 mental activities were selected as the best strategies for the second part. The second part was completed on day 4 after completing the first part. It was composed of four fixed sessions. Each session contained 12 60-second trials and an interval of 10 seconds between two successive trials. The total duration of the second part was almost one hour. The patient applied the most effective mental strategies selected from the user control sessions for training, but only one was used for each trial. Each strategy was repeated twice in each fixed session.

### 2.3. Feedback Display

The display showed a sphere and a cube. The sphere reflected the feedback parameter value. If it reached a predefined threshold (goal 1), the sphere color changed from white to purple. This sphere was constituted by several slices, and the more slices it had, the smoother it looked. Initially, the sphere was only constituted by four slices, which was the minimum number. While goal 1 was being achieved, the slices were slowly added to the sphere. Otherwise, the sphere loosed slice slowly until it only had four slices again. The cube height was related to the period of time for which goal 1 kept being achieved continuously. If goal 1 was being achieved continuously for more than 2 seconds, goal 2 was accomplished and the cube rose up until goal 1 stopped being achieved. Then the cube started falling slowly until it reached the bottom, or goal 2 was achieved again. Thus, the best outcome was to keep the cube as high as possible.

The threshold was set to 1 in the first user control session, and it could be adjusted in each session. A report was generated after every session, which showed the percentage of time for the feedback parameter above the threshold. If this value exceeded 60%, the threshold was increased by 0.1 in the next session. In contrast, if this value was lower than 30%, the threshold was decreased by 0.1 in the next session.

### 2.4. Short-Term Memory Test

Short-term memory performance was assessed by the forward and backward digit span before and at the end of the NF training. Each test was composed of a series of trials demonstrating random digits at the rate of one digit per second. At each trial, the number of digits shown was increased by one until the patient failed twice to recollect every digit. The last number of digits correctly recollected was the patient's digit span score. In each test, after all the digits had been shown, the patient was instructed to enter the digits with the same order (forward digit span) or with the inverse order (backward digit span) as they were presented.

### 2.5. Data Analysis

After all the training, the mean relative amplitude (relative to 0.5–30 Hz) in each session was calculated in not only the training frequency bands: the IAB and the beta2 (20–30 Hz), but also the neighboring frequency bands: the individual theta (4 Hz-LTF), the individual sigma (HTF-16 Hz), and the beta1 (16–20 Hz).

## 3. Results

### 3.1. EEG

**Figure 2:**
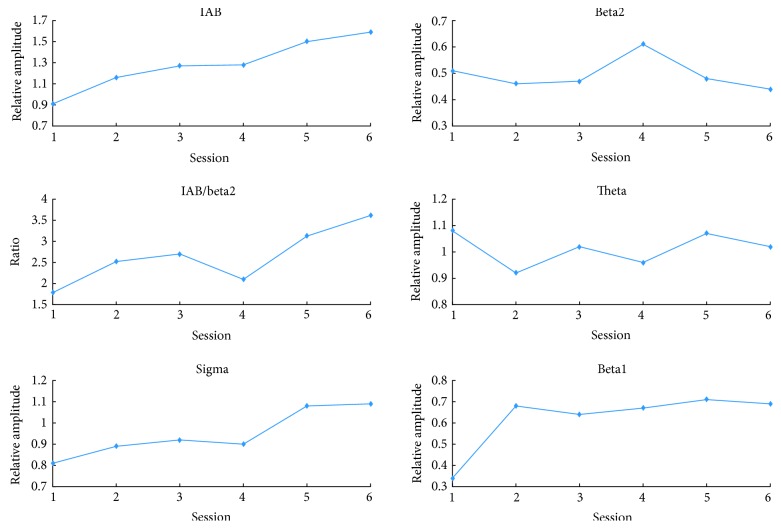
EEG results during NF sessions. Session 1 and session 2 are the user control sessions. Session 3 to session 6 are the fixed sessions.

Different from the standard alpha band (8–12 Hz), the IAB was between 5.8 and 10.8 Hz and the peak alpha frequency was 9.5 Hz. Thus, the theta band ranged from 4 Hz to 5.8 Hz and the sigma band ranged from 10.8 Hz to 16 Hz. Since the duration varied across the user control sessions, we only presented the first two user control sessions and the four fixed sessions to examine the EEG change from the beginning to the end of training. In line with the training objective, the relative IAB amplitude shows an increase trend while the relative beta2 amplitude presents a decrease trend along sessions (see Figure 2). Specially, the relative IAB amplitude increased 74.73% while the relative beta2 amplitude decreased 13.73% from the first user control session to the last fixed session. We can see that the IAB obtained much larger change than the beta2, indicating that self-regulation of IAB was easier than that of the beta2 for this patient. In addition, the feedback parameter IAB/beta2 increased from 1.78 to 3.61. The above results indicated that the subject could learn to self-regulate the requested brain activity by the NF training. Besides the training frequency bands, other frequency bands also demonstrated some changes shown in Figure 2. Theta did not have obvious change trend over sessions while the sigma and the beta1 showed an increase trend over sessions.

The threshold obtained gradual increase over sessions (from 1 to 3.5), which also indicated that the patient could overcome the difficulties of training and achieve a high value of the training parameter. In the last several sessions, the largest percentage time of exceeding threshold 3.5 was 57.64%.

### 3.2. Effective Mental Strategy

During the user control sessions, the patient applied different types of mental strategies such as family members, friends, school life, previous job, bodhisattva, sutra, and natural scenery. During the session intervals, she read books and talked with a psychotherapist who accompanied her while the EEG was recorded. The conversation included her frequent thoughts about suicidal ideation, hatred, family issues, future (worry), complaints, abnormal behavioral patterns (such as getting naked), and so on since her first noticeable episode. The psychotherapist tried to calm down negative mood and change her negative thinking. By the user control sessions, it was found that the most effective mental strategies were natural scenery including waterfall, moon, lake, mountain, lotus, and sea.

### 3.3. Behavioral Performance

For the short-term memory test, the forward digit test was 7 and the backward digit test was 5 before training. At the end of training, the forward digit test was 9 and the backward digit test was 6. The patient reported that it was easier to remember the digits after training. Moreover, the patient showed large improvement in mood and speech pattern scored by her psychotherapist.

Before NF training, the patient had positive and negative psychotic behavioral symptoms which were not shown in the healthy people. After the end of NF training, she kept on practicing the most effective training strategies at home daily without any NF training monitor administered. We used a scale (0–9), where “0” represents the least noticeable symptoms and “9” represents the most noticeable symptoms. The participant's positive and negative symptoms dramatically improved on the 22 months follow-up. As shown in Table 1 scored by her psychotherapist, most positive and negative symptoms were least noticeable after 22 months of completing NF. Nowadays, she is back to the community and working as a home health aide in taking care of the elderly.

## 4. Discussion

The objective of this study was to explore the effect of short but intensive NF training for patient with chronic schizophrenia. A middle-aged woman with chronic schizophrenia participated in the experiment. According to the symptoms of this patient, the abnormal alpha and beta activities in schizophrenics [12–15], Bolea's clinical experience [8], and the antipsychotic treatment and TMS for schizophrenia [16, 17], the intensive NF training was designed to increase alpha amplitude simultaneously to decrease the beta2 amplitude at P4 location.

From the training duration of each day (more than 1 hour on day 1 and more than 3 hours on the remaining days), it was observed that the patient was willing to perform such intensive training. In spite of only four days of NF, the patient found out the most effective mental strategies and learnt how to regulate her brain activity. Consistent with our training objective, the relative IAB amplitude increased and relative beta2 amplitude decreased over training sessions. Although the patient maintained the medications during the training period, we speculated that her EEG changes mainly resulted from the NF training for two major reasons. First, since she already took medications before training for a long time, the contribution from medications became relatively stable. Observed changes of the EEG during training were very likely due to NF training. Second, the patient found out effective mental strategies to regulate her alpha and beta2 activities during the NF training, and such increase in alpha and decrease in beta2 were in line with the NF setting.

The above EEG changes by NF training were associated with her observable cognitive and behavioral improvements, such as short-term memory, mood, and speech patterns after training. What is more, with the follow-up study after 22 months of completing NF, she showed a dramatic improvement in both positive and negative symptoms, displayed a remarkable progress, and was treated as a capable person. Similarly, we think that these positive outcomes resulted from the NF training, which could be explained from the following two aspects. First, the patient had no noticeable improvement for more than seven years with either single antipsychotic treatment or combined antipsychotic and psychotherapeutic treatment before NF training. Second, she learnt how to regulate her brain activities through the NF training, which enhanced her confidence in self-control. From her psychotherapist's report, although no more NF training was conducted after her first NF training, she still practiced useful mental strategies daily at home, at least, up until 22 months later from her first NF training. By inference, therefore, her improvement was due to the NF training since there were no major differences with the patient's other treatments, psychotic medications, and psychotherapy.

Bolea [8] suggested that reduction of fast beta and enhancement of alpha (8–12 Hz) at P4, PO4, and CP4 seemed very important for chronic schizophrenia patient, and our study partially confirmed this aspect. The patient achieved a reduction of the beta2 and an enhancement of the IAB at P4 by only four days and improved her symptoms dramatically in the follow-up data. To some extent, our result is also consistent with the finding that the enhancement of the alpha power by TMS led to the significant reduction in negative symptoms in the patients with schizophrenia [17].

This study presented a preliminary step to apply short but intensive NF training on the schizophrenic patient with auditory hallucinations. The limitations in this work include small patient number, single channel EEG recording, and the lack of follow-up EEG data. The change of EEG at other locations and the lasting effects on EEG are not clear yet. Moreover, there are six subtypes of schizophrenics and each subtype is associated with a different electrophysiological profile [21]. Future study shall include a larger number of patients, multichannel EEG measurements, and control groups in order to fully examine the effects of such NF training on all aspects.

In conclusion, the patient with schizophrenia showed a dramatic improvement by the intensive NF training of alpha/beta2. This relatively short but intensive NF training protocol may offer a promising therapeutic approach for schizophrenia and thus deserves further study to fully examine its scope.

## Figures and Tables

**Table 1:** Symptom comparison.

Symptom type	Symptoms	Pre-NF training	22nd-month follow-up
Positive	Visual hallucination	7	0
	Auditory hallucination	9	1
	False beliefs	9	1~2
	Distorted reasoning	9	1
	Disorganized speech	9	0
	Misinterpreted perception	9	0~1

Negative	Limited facial expression	9	0
	No emotional and verbal communication	8	0
	Restricted body movement and gesture	9	0
	No motivation to initiate things	8	0~1
